# Influence of Roasting Condition on Flavor Profile of Sunflower Seeds: A flavoromics approach

**DOI:** 10.1038/s41598-019-47811-3

**Published:** 2019-08-05

**Authors:** Shuangshuang Guo, Kriskamol Na Jom, Yan Ge

**Affiliations:** 1 Department of Food Science and Technology, Faculty of Agro-Industry, Kasetsart University, Bangkok, 10900 Thailand; 2 The Academy of Science, Nanjing Agricultural University, Nanjing, 210095 The People’s Republic of China

**Keywords:** Process chemistry, Gas chromatography

## Abstract

Sunflower see/ds (*Helianthus annuus* L.) were roasted in an electric forced air oven for 15, 30, 45, and 60 min at 125, 135 and 145 °C. The effect of temperature and time on the flavor profile of the samples were evaluated by headspace solid-phase microextraction coupled with gas chromatography-mass spectroscopy (HS-SPME-GC-MS). Unsupervised Principle Component Analysis (PCA) and Agglomerative Hierarchical Clustering (AHC) multivariate statistical methods were used to visualize, group and classify the samples. 114 volatiles were identified in the roasted sunflower seeds (RSF), with terpenes (α-pinene, β-pinene), heterocyclic compounds (2-ethyl-3-methylpyrazine, 2,5-dimethylpyrazine, 2-ethyl-3,5-dimethylpyrazine, pyridine), aldehydes (2-methylbutanal, furfural, hexanal, phenylacetaldehyde), hydrocarbons (octane, 2-isobutyl-1,4-dimethylcyclohexane, 6,6-dimethylundecane), alcohol (3-methyl-2-propyl-1-pentanol), and γ-butyrolactone being dominant compounds. The content of most volatile compounds increased with increase in roasting temperature and time, such as esters, terpenes, pyrazines, aldehydes, ketones, and alcohols. 2,3-dimethylpyrazine, 2,5-dimethylpyrazine, 2-ethyl-3-methylpyrazine, and 2-ethyl-3,5-dimethylpyrazine contributed to be the major role in roast and nutty flavor of the roasted sunflower seeds. Roasting at 125 °C for 45 min was found to be the better condition for roasted sunflower seeds, which gave the lowest off-flavor and burnt tastes.

## Introduction

Sunflower seed is a kind of food with a large number of nutrients including unsaturated fat, protein, polyphenols, vitamins, and minerals. They are used in many food applications, such as edible oil or snack and more^1^. In comparison to other oilseeds, sunflower seeds have approximately 31% of polyunsaturated fatty acids, which is 28.2%, 25.5%, 22.4%, 18.1%, 13.1%, and 3.5% in safflower seed, sesame, flax, cottonseed, peanut, and soy, respectively. The roasting process is a kind of preparation method and heat treatment used for the development of more desirable aroma and taste, as well as reduces or eliminates microbes according to food safety standards. Roasting involves a number of physicochemical changes including dehydration and chemical reactions^2^. The aroma derived from the Maillard reaction is liked by the consumers. On the contrary, the high temperature for roasting may result in reduced nutrients or undesirable aroma in the kernels. In addition, the sensitivity of unsaturated fatty acids to oxidation depends directly on the degree of unsaturation^3,4^. Therefore, fatty acid composition makes sunflower seeds susceptible to deteriorate during roasting and storage. Low molecular weight compounds produced in the oxidation process can lead to off-flavor in the seeds, making the seeds unacceptable to consumers, and also can not be used as industrial or food ingredients^5^. Thus it is necessary to optimum roasting temperature and time in addition to desirable flavor.

Flavoromics is an “-omic” and “-holistic” approach focused on low molecular mass compounds (volatile and non-volatile) and linking them to a defined sensorial perception^6^. Flavoromics is mainly applied to indicate changes in chemical compositions during roasting and storage. GC-MS is a useful technology mainly applied for food flavor analysis in recent years^7,8^. Quantitative analysis of food volatiles by HS coupled with GC-MS has the advantages of fast, simple, eliminating column degradation, nonvolatile residues and nondestructive. In particular, SPME represents an especially promising sample preparation technique in food analysis for the pre-processing procedures of GC-MS^9^. Multiple samples can be collected without obvious composition changes in the initial product. SPME is a useful extraction technique using a thin silica whisker coated with chromatographic material that can be introduced into the HS or directly into the liquid samples^10^. In this method, volatiles substances are directly adsorbed on adsorbents located on fused-silica fiber and then thermal desorption is carried out at the inlet of GC. HS-SPME-GC-MS has become the most important technology in flavoromics and been used to quantitatively and qualitatively measure large-scale volatile compounds in complex samples^9,11,12^. However, No information has been reported about the application of HS-SPME-GC-MS for studying the flavor profile of roasted sunflower seeds. PCA and AHC analysis are necessary for spectral analysis which is an effective variable reduction technique for spectroscopic data used to differentiate sample classes. This study aims to use flavoromics approach to study the impact a wide range of roasting process on the flavor profile of sunflower seed.

## Results and Discussion

### Flavoromic profile of the roasted sunflower seed

In this study, HS-SPME-GC-MS method was applied for extraction and determination of the volatile compounds in the roasted sunflower seeds during roasting. Desorption time 20 min was selected according to the preliminary experiment because higher desorption time showed high desorption efficiencies of all analytes, in addition, increasing desorption time prevented the cross-contamination with the previous sample. Table 1 showed the major identified volatile compounds exist in the HS of the roasted sunflower seeds. 114 aroma compounds were identified within the roasted sunflower seeds samples. They belong to the chemical classes of ketones, aldehydes, pyrazines, alcohols, pyrroles, easters, furan derivatives, heterocyclic compounds, as well as hydrocarbons. Table 2 listed the key odor compounds existed in the roasted sunflower seeds. During the roasting process, A large number of volatile compounds are formed by Maillard reaction and lipid oxidation, including aldehydes, alcohols, ketones, pyrazines, furans. Pyrazines contributed to nutty and roasted flavor. Furan-containing compounds are also possibly produced from the thermal degradation of sugars such as fructose and glucose^13^. Aldehydes and alcohols are formed from lipid oxidation, such as nonanal, hexanal. Nonanal contributes to tallow and fruity flavor, 1-octen-3-ol is generated from thermal decomposition of methyllinoleate hydroperoxide, may contribute to an herbaceous aroma. The concentration (μg/g) of volatile components in sunflower seeds roasted from 125 to 145 °C for 15, 30, 45 and 60 min presented in Table 1.

**Table 1:** Concentrations (μg/g) of volatile flavor components in sunflower seeds roasted from 125 to 145 °C for 15, 30, 45 and 60 min.

Components	RI	0	Relative concentration (μg/g)											
			125 °C				135 °C				145 °C			
			15 m	30 m	45 m	60 m	15 m	30 m	45 m	60 m	15 m	30 m	45 m	60 m
Isobutanal	629	0.21 ± 0.06	0.22 ± 0.06	0.10 ± 0.03	0.17 ± 0.04	—	0.24 ± 0.06	0.35 ± 0.09	—	0.36 ± 0.05	—	—	0.48 ± 0.06	1.22 ± 0.16
3-Methylbutanal	646	0.28 ± 0.07	0.11 ± 0.03	0.21 ± 0.06	0.31 ± 0.08	1.60 ± 0.43	—	—	0.19 ± 0.03	0.31 ± 0.04	—	0.18 ± 0.02	0.26 ± 0.04	0.74 ± 0.10
2-Methylbutanal	662	0.54 ± 0.14	0.21 ± 0.06	0.27 ± 0.07	—	—	—	0.55 ± 0.15	0.37 ± 0.05	0.77 ± 0.10	0.03 ± 0.01	0.25 ± 0.03	0.77 ± 0.10	2.11 ± 0.28
3-Penten-2-one	711	0.45 ± 0.12	0.14 ± 0.04	0.04 ± 0.01	0.32 ± 0.09	0.19 ± 0.05	0.38 ± 0.10	0.59 ± 0.16	0.25 ± 0.03	0.42 ± 0.06	—	0.23 ± 0.03	0.23 ± 0.03	0.67 ± 0.09
Pyridine	717	0.11 ± 0.03	0.41 ± 0.11	1.51 ± 0.41	1.19 ± 0.32	3.13 ± 0.84	0.31 ± 0.08	—	0.74 ± 0.10	2.66 ± 0.36	—	0.92 ± 0.12	2.81 ± 0.38	2.70 ± 0.36
Toluene	751	0.24 ± 0.07	0.33 ± 0.09	0.15 ± 0.04	0.29 ± 0.08	0.27 ± 0.07	0.18 ± 0.05	0.19 ± 0.06	0.36 ± 0.05	0.40 ± 0.05	0.17 ± 0.02	0.17 ± 0.02	0.18 ± 0.03	0.63 ± 0.08
Hexanal	757	1.35 ± 0.36	0.48 ± 0.13	0.68 ± 0.18	1.35 ± 0.36	3.35 ± 0.09	0.92 ± 0.25	1.74 ± 0.47	1.00 ± 0.13	1.96 ± 0.26	0.56 ± 0.08	0.81 ± 0.11	2.50 ± 0.34	8.49 ± 1.14
Octane	796	2.67 ± 0.72	1.28 ± 0.34	0.13 ± 0.03	0.54 ± 0.15	5.50 ± 1.48	0.91 ± 0.24	1.77 ± 0.47	0.60 ± 0.08	1.15 ± 0.16	0.21 ± 0.03	1.09 ± 0.15	—	—
Furfural	800	0.95 ± 0.25	0.35 ± 0.10	0.57 ± 0.15	0.87 ± 0.23	2.98 ± 0.80	0.42 ± 0.11	1.92 ± 0.52	1.21 ± 0.16	3.46 ± 0.47	0.53 ± 0.07	3.63 ± 0.49	5.09 ± 0.68	8.18 ± 1.10
2,3,3-Trimethyl-1,4-pentadiene	805	—	0.17 ± 0.05	0.12 ± 0.03	0.34 ± 0.09	1.21 ± 0.32	0.30 ± 0.08	0.87 ± 0.23	0.41 ± 0.06	0.89 ± 0.12	—	—	0.85 ± 0.11	1.97 ± 0.26
(Z)-2-Octene	810	—	0.95 ± 0.25	0.04 ± 0.01	0.26 ± 0.07	0.59 ± 0.16	0.16 ± 0.04	0.61 ± 0.16	0.35 ± 0.05	0.67 ± 0.09	0.06 ± 0.01	—	0.75 ± 0.10	1.07 ± 0.14
2,6-Dimethylheptane	828	0.67 ± 0.18	—	—	—	1.34 ± 0.36	—	—	0.25 ± 0.03	0.86 ± 0.12	0.03 ± 0.12	0.12 ± 0.02	0.40 ± 0.05	1.02 ± 0.14
Ethylcyclohexane	830	0.54 ± 0.13	0.24 ± 0.06	0.22 ± 0.06	0.41 ± 0.11	1.72 ± 0.46	0.33 ± 0.09	0.68 ± 0.18	0.40 ± 0.05	0.85 ± 0.11	0.08 ± 0.01	0.25 ± 0.03	0.56 ± 0.07	1.63 ± 0.22
1,1,3-Trimethylcyclohexane	835	0.44 ± 0.12	0.20 ± 0.04	0.15 ± 0.04	0.34 ± 0.09	1.52 ± 0.41	0.27 ± 0.07	0.49 ± 0.13	0.33 ± 0.04	0.67 ± 0.09	0.06 ± 0.01	0.21 ± 0.03	0.42 ± 0.06	1.30 ± 0.17
n-Hexanol	849	1.94 ± 0.52	1.84 ± 0.50	1.23 ± 0.33	0.31 ± 0.08	0.20 ± 0.05	0.85 ± 0.23	0.63 ± 0.17	0.35 ± 0.05	0.56 ± 0.07	1.04 ± 0.14	0.16 ± 0.02	0.29 ± 0.04	0.04 ± 0.01
1,3,5-Trimethylcyclohexane	850	0.83 ± 0.22	0.30 ± 0.08	0.04 ± 0.01	—	1.11 ± 0.30	—	0.49 ± 0.13	0.32 ± 0.04	0.57 ± 0.08	—	0.15 ± 0.02	0.37 ± 0.05	0.97 ± 0.13
γ-Butyrolactone	850	1.15 ± 0.31	0.45 ± 0.12	0.30 ± 0.08	0.90 ± 0.24	2.25 ± 0.60	0.56 ± 0.15	1.36 ± 0.37	0.78 ± 0.10	1.72 ± 0.23	0.09 ± 0.01	0.37 ± 0.05	1.43 ± 0.19	3.19 ± 0.43
Methional	854	—	—	0.13 ± 0.04	0.16 ± 0.04	0.59 ± 0.16	0.34 ± 0.09	0.42 ± 0.11	0.54 ± 0.07	0.64 ± 0.08	0.17 ± 0.02	0.39 ± 0.05	0.51 ± 0.07	0.64 ± 0.09
2-Methyloctane	863	0.47 ± 0.13	0.21 ± 0.06	—	0.36 ± 0.10	1.26 ± 0.34	—	—	0.33 ± 0.04	0.73 ± 0.10	0.06 ± 0.01	0.29 ± 0.04	0.36 ± 0.05	0.97 ± 0.15
2-Heptanone	866	—	0.62 ± 0.17	—	—	0.42 ± 0.11	—	—	—	0.24 ± 0.03	—	—	0.23 ± 0.03	0.54 ± 0.07
3-Methyloctane	870	0.26 ± 0.07	0.13 ± 0.04	0.05 ± 0.01	0.24 ± 0.06	0.81 ± 0.22	0.19 ± 0.05	0.34 ± 0.09	0.20 ± 0.03	0.49 ± 0.07	0.04 ± 0.00	0.14 ± 0.02	0.29 ± 0.04	0.96 ± 0.13
2,5-Dimethylpyrazine	885	0.20 ± 0.05	0.10 ± 0.03	0.99 ± 0.27	0.98 ± 0.28	1.71 ± 0.46	0.49 ± 0.13	2.47 ± 0.66	0.81 ± 0.11	2.56 ± 0.34	0.52 ± 0.07	0.54 ± 0.07	5.17 ± 0.69	10.19 ± 1.37
n-Nonane	899	0.28 ± 0.07	0.12 ± 0.03	0.06 ± 0.02	0.22 ± 0.06	0.70 ± 0.19	0.18 ± 0.05	0.37 ± 0.10	0.16 ± 0.02	0.51 ± 0.07	0.03 ± 0.00	0.14 ± 0.02	0.33 ± 0.04	0.96 ± 0.13
Benzaldehyde	926	0.35 ± 0.09	0.06 ± 0.02	0.11 ± 0.03	0.16 ± 0.04	0.60 ± 0.16	0.17 ± 0.04	0.63 ± 0.17	0.36 ± 0.05	0.40 ± 0.05	—	0.08 ± 0.01	0.87 ± 0.12	0.75 ± 0.10
α-Pinene	930	7.57 ± 2.03	5.82 ± 1.56	7.05 ± 1.89	12.50 ± 3.4	12.70 ± 3.4	6.85 ± 1.84	10.79 ± 2.9	6.56 ± 0.88	13.71 ± 1.84	1.18 ± 0.16	4.72 ± 0.63	10.20 ± 1.4	21.85 ± 2.94
Camphene	943	0.34 ± 0.09	0.29 ± 0.08	0.06 ± 0.02	0.48 ± 0.13	0.20 ± 0.05	0.28 ± 0.08	0.41 ± 0.11	0.28 ± 0.04	0.53 ± 0.07	0.04 ± 0.01	0.17 ± 0.02	0.36 ± 0.05	0.96 ± 0.13
Thujane-2,4(10)-diene	946	—	—	0.04 ± 0.01	—	0.61 ± 0.16	0.07 ± 0.02	—	—	—	—	0.04 ± 0.00	0.14 ± 0.02	0.35 ± 0.05
Phenol	951	0.27 ± 0.07	0.14 ± 0.04	0.08 ± 0.02	0.25 ± 0.07	0.55 ± 0.15	0.14 ± 0.04	0.24 ± 0.06	0.11 ± 0.01	0.29 ± 0.04	—	0.06 ± 0.01	—	—
1-Octen-3-ol	959	0.27 ± 0.06	0.14 ± 0.04	0.06 ± 0.02	—	—	0.25 ± 0.07	0.27 ± 0.06	0.17 ± 0.02	—	0.44 ± 0.06	0.38 ± 0.05	0.06 ± 0.01	—
β-Phellandrene	965	0.24 ± 0.07	0.22 ± 0.06	0.15 ± 0.04	0.39 ± 0.10	1.83 ± 0.49	0.26 ± 0.07	0.48 ± 0.13	0.20 ± 0.03	0.52 ± 0.07	0.04 ± 0.01	0.13 ± 0.02	0.37 ± 0.05	0.80 ± 0.11
3-Methylnonane	968	0.13 ± 0.03	0.10 ± 0.03	0.04 ± 0.01	0.14 ± 0.04	0.50 ± 0.13	0.10 ± 0.03	0.19 ± 0.05	0.10 ± 0.01	0.26 ± 0.04	0.02 ± 0.00	0.05 ± 0.01	—	—
β-Pinene	970	0.76 ± 0.20	0.49 ± 0.13	0.16 ± 0.04	0.98 ± 0.26	1.66 ± 0.45	0.58 ± 0.16	0.85 ± 0.23	0.53 ± 0.07	1.15 ± 0.15	0.08 ± 0.01	0.37 ± 0.05	1.25 ± 0.17	3.00 ± 0.40
2-Ethyl-3-methylpyrazine	974	0.14 ± 0.04	0.13 ± 0.03	0.36 ± 0.10	0.54 ± 0.15	0.88 ± 0.24	0.14 ± 0.03	1.63 ± 0.44	2.27 ± 0.31	2.86 ± 0.38	—	0.18 ± 0.02	1.96 ± 0.26	3.89 ± 0.52
2-Pentylfuran	976	0.33 ± 0.09	0.89 ± 0.24	0.21 ± 0.06	0.14 ± 0.04	1.53 ± 0.41	0.18 ± 0.05	0.54 ± 0.14	0.13 ± 0.02	0.30 ± 0.04	—	0.07 ± 0.01	0.53 ± 0.07	1.17 ± 0.16
3(E)-3-methyl-3-nonene	981	0.18 ± 0.05	0.10 ± 0.03	0.07 ± 0.02	0.16 ± 0.04	0.58 ± 0.15	0.14 ± 0.04	0.29 ± 0.08	0.13 ± 0.02	0.31 ± 0.04	0.02 ± 0.01	0.07 ± 0.01	0.16 ± 0.02	0.74 ± 0.10
Pantoic lactone	987	0.12 ± 0.03	0.26 ± 0.07	0.05 ± 0.01	0.06 ± 0.02	—	0.04 ± 0.01	0.14 ± 0.04	0.07 ± 0.01	0.19 ± 0.03	—	—	0.32 ± 0.04	0.49 ± 0.07
4-Decene	989	0.32 ± 0.09	0.22 ± 0.06	0.18 ± 0.05	0.34 ± 0.09	—	0.26 ± 0.07	0.51 ± 0.14	0.26 ± 0.04	1.03 ± 0.14	0.06 ± 0.01	0.22 ± 0.03	0.56 ± 0.08	1.84 ± 0.25
Decane	1000	0.34 ± 0.08	0.21 ± 0.06	0.11 ± 0.03	0.35 ± 0.09	0.89 ± 0.24	0.23 ± 0.05	0.52 ± 0.14	0.21 ± 0.02	0.60 ± 0.08	0.05 ± 0.01	0.14 ± 0.02	0.36 ± 0.05	0.97 ± 0.13
Phenylacetaldehyde	1007	0.27 ± 0.07	0.22 ± 0.06	0.34 ± 0.09	0.77 ± 0.21	1.26 ± 0.22	0.20 ± 0.05	0.47 ± 0.13	0.13 ± 0.02	0.27 ± 0.04	0.68 ± 0.09	0.95 ± 0.13	1.25 ± 0.17	1.29 ± 0.17
p-Cymene	1011	0.11 ± 0.03	0.67 ± 0.18	0.05 ± 0.01	—	0.37 ± 0.10	0.09 ± 0.02	0.23 ± 0.06	0.07 ± 0.01	0.16 ± 0.02	—	—	0.33 ± 0.04	0.70 ± 0.09
D-Limonene	1020	0.27 ± 0.07	0.25 ± 0.07	0.16 ± 0.04	0.28 ± 0.08	1.15 ± 0.31	0.16 ± 0.04	0.44 ± 0.12	0.20 ± 0.03	0.47 ± 0.06	0.03 ± 0.01	0.10 ± 0.01	0.17 ± 0.02	0.39 ± 0.05
1,4-Butanediol diacetate	1041	0.92 ± 0.25	0.15 ± 004	0.15 ± 0.04	0.89 ± 0.24	0.71 ± 0.18	0.38 ± 0.10	1.26 ± 0.30	0.49 ± 0.07	1.50 ± 0.20	—	0.22 ± 0.03	0.69 ± 0.09	1.68 ± 0.23
2-Ethyl-3,5-dimethylpyrazine	1053	—	—	0.19 ± 0.05	0.19 ± 0.04	0.36 ± 0.10	0.08 ± 0.02	0.30 ± 0.08	0.12 ± 0.02	0.53 ± 0.07	—	0.07 ± 0.01	1.11 ± 0.15	2.09 ± 0.28
N-allyl- cyclopentane-carboxamide	1056	1.52 ± 0.41	—	0.58 ± 0.16	1.37 ± 0.37	—	0.96 ± 0.26	1.71 ± 0.46	1.02 ± 0.14	—	0.33 ± 0.04	0.67 ± 0.09	—	—
2,4-Dimethyl-1-decene	1065	0.25 ± 0.07	0.06 ± 0.02	0.12 ± 0.03	0.23 ± 0.06	0.89 ± 0.24	0.17 ± 0.04	0.27 ± 0.07	0.17 ± 0.02	0.57 ± 0.08	0.06 ± 0.01	0.11 ± 0.02	0.38 ± 0.05	1.01 ± 0.14
Nonanal	1081	0.32 ± 0.09	0.28 ± 0.08	0.21 ± 0.06	0.51 ± 0.14	0.79 ± 0.21	0.31 ± 0.08	0.24 ± 0.06	0.54 ± 0.07	0.63 ± 0.08	0.39 ± 0.05	0.44 ± 0.06	0.56 ± 0.07	1.06 ± 0.14
6-Ethyl-3-octanone	1083	0.38 ± 0.10	—	—	—	1.25 ± 0.34	0.27 ± 0.07	0.48 ± 0.13	0.26 ± 0.03	0.73 ± 0.10	0.08 ± 0.01	0.16 ± 0.03	0.39 ± 0.05	1.19 ± 0.16
2,6-Dimethy-lundecane	1120	—	0.43 ± 0.11	—	—	1.64 ± 0.45	—	—	—	1.09 ± 0.15	—	—	0.54 ± 0.07	3.97 ± 0.53
6,6-Dimethy-lundecane	1140	0.42 ± 0.11	0.47 ± 0.13	0.22 ± 0.06	0.44 ± 0.12	1.99 ± 0.53	0.44 ± 0.12	0.84 ± 0.22	0.35 ± 0.05	1.26 ± 0.17	0.08 ± 0.01	0.19 ± 0.03	0.65 ± 0.09	1.94 ± 0.26
5-Methylundecane	1157	0.28 ± 0.08	0.21 ± 0.06	0.10 ± 0.02	0.39 ± 0.10	0.74 ± 0.20	0.27 ± 0.07	0.66 ± 0.18	0.19 ± 0.02	0.51 ± 0.07	0.07 ± 0.01	0.09 ± 0.01	0.26 ± 0.02	0.74 ± 0.10
3-Methylundecane	1171	—	0.19 ± 0.05	—	—	0.68 ± 0.18	—	—	—	0.48 ± 0.06	—	—	0.26 ± 0.03	0.70 ± 0.09
1-Dodecene	1189	0.27 ± 0.07	0.20 ± 0.04	0.13 ± 0.03	0.17 ± 0.05	0.45 ± 0.12	0.27 ± 0.07	0.58 ± 0.16	0.19 ± 0.03	0.49 ± 0.07	0.07 ± 0.02	0.010 ± 0.01	0.26 ± 0.03	0.67 ± 0.09
Dodecane	1201	0.35 ± 0.10	0.25 ± 0.07	0.13 ± 0.04	0.34 ± 0.09	0.73 ± 0.19	0.28 ± 0.08	0.65 ± 0.19	0.20 ± 0.03	0.55 ± 0.07	0.08 ± 0.01	0.13 ± 0.02	0.31 ± 0.04	0.80 ± 0.11
3-Methyltridecane	1368	—	—	—	—	—	—	0.09 ± 0.02	0.10 ± 0.01	—	—	—	—	—
1-Tetradecene	1388	0.14 ± 0.04	0.12 ± 0.03	0.07 ± 0.02	0.14 ± 0.04	0.25 ± 0.07	0.13 ± 0.03	0.40 ± 0.11	0.10 ± 0.01	0.31 ± 0.04	0.07 ± 0.01	0.12 ± 0.02	0.13 ± 0.01	0.44 ± 0.06
Tetradecane	1399	0.13 ± 0.03	0.61 ± 0.16	0.06 ± 0.02	0.02 ± 0.00	—	0.02 ± 0.01	0.19 ± 0.05	0.09 ± 0.01	0.03 ± 0.00	—	0.01 ± 0.00	0.03 ± 0.01	0.31 ± 0.04
Octyl methacrylate	1424	0.18 ± 0.03	0.12 ± 0.01	-	0.17 ± 0.05	0.33 ± 0.09	0.16 ± 0.04	0.35 ± 0.09	0.11 ± 0.01	0.29 ± 0.04	0.06 ± 0.01	0.06 ± 0.01	0.14 ± 0.02	0.40 ± 0.04
7-Methyltridecane	1429	0.20 ± 0.05	0.14 ± 0.04	0.08 ± 0.02	0.16 ± 0.04	0.38 ± 0.10	0.17 ± 0.05	0.40 ± 0.11	0.12 ± 0.02	0.33 ± 0.04	0.06 ± 0.01	0.06 ± 0.02	0.116 ± 0.02	0.41 ± 0.05
Calarene	1433	0.31 ± 0.08	0.25 ± 0.07	0.12 ± 0.03	0.33 ± 0.09	0.62 ± 0.17	0.28 ± 0.06	0.57 ± 0.15	0.20 ± 0.03	0.42 ± 0.06	0.08 ± 0.01	0.08 ± 0.01	0.24 ± 0.02	0.70 ± 0.09
Chamigrene	1456	0.12 ± 0.03	—	—	0.12 ± 0.03	—	0.10 ± 0.03	0.20 ± 0.05	0.07 ± 0.01	—	0.01 ± 0.00	—	—	—
5,6-Dipropyldecane	1465	0.46 ± 0.12	—	0.18 ± 0.05	0.39 ± 0.10	—	0.37 ± 0.10	0.86 ± 0.23	0.27 ± 0.04	—	0.15 ± 0.02	0.14 ± 0.02	—	—
β-Bisabolene	1499	0.27 ± 0.07	0.25 ± 0.06	0.03 ± 0.01	0.33 ± 0.09	—	0.31 ± 0.08	0.42 ± 0.11	0.20 ± 0.03	0.27 ± 0.04	0.05 ± 0.01	0.07 ± 0.01	0.21 ± 0.03	0.48 ± 0.06

Not detected.

**Table 2:** Key odorants content obtained from roasted ungerminated and germinated sunflower seed which was roasted at 125, 135 and 145 °C for 15, 30, 45 and 60 min.

Compounds	Odor quality^a^	Odor threshold^b^ (μg/g)	Roasted SF seeds
**Pyrazines**			
2,5-Dimethylpyrazine	Roasty, flowery, cocoa	0.8	7,9,12,13
2,3-Dimethylpyrazine	Nut, peanut, cocoa, meat	0.1	12,13
3-Ethyl-2,5-dimethylpyrazine	Potato, roast	0.079	N
2-Ethyl-3-methylpyrazine	Nutty, cereal like	0.02	1,2,3,4,5,6,7,8,9,11,12
2-Ethyl-3,5-dimethylpyrazine	Nutty	0.0075	3,4,5,6,7,8,9,11,12,13
**Aldehydes**			
2-Methylbutanal	Cocoa, almond	0.023	1,2,3,7,8,9,10,11,12,13
3-Methylbutanal	Malt	0.0108	1,2,3,5,8.9.11,12,13
Hexanal	Green, fatty	0.479	1,2,3,4,5,6,7,8,9,10,11,12,13
Furfural	Bread, almond, sweet	3	9,11,12,13
Heptanal	Fat, citrus, racid	0.05	N
Nonanal	Fatty, green	0.26	1,2,4,5,6,8,9,10,11,12,13
Benzaldehyde	Almond, sugar	3.6	N
Phenylacetaldehyde	Flowery, honey like	0.022	1,2,3,4,5,6,7,8,9,10,11,12,13
**Ketones**			
2-Heptanone	Soap	1.5	N
ɣ-Butyrolactone	Creamy	1	1,5,7,9,12,13
Alcohols			
1-Pentanol	Balsamic	0.47	5
1-Hexanol	Resin, flower, green	0.4	1,2,3,6,7,9,10
Furfuryl alcohol	Sweet, bread-like, caramellic	2	N
Pyridine	Burnt, smoky	2	5,9,12,13
**Sulfur compounds**			
Dimethyl disulfide	Onion, cabbage, putrid	0.00006	N
Methional	Cooked, potato	0.00005	3,4,5,6,7,8,9,10,11,12,13
Others			
α-Pinene	Pine, turpentine	0.006	1,2,3,4,5,6,7,8,9,10,11,12,13
2-Acetylfuran	Peanut, sweet	15.025	N

^a^Odor quality is obtained according to the website: http://www.flavornet.org/flavornet.html.

^b^Orthonasal odor threshold values are according to literature^23,31–35^.

SF number representation: 1 (raw seed), 2 (125 °C for 15 min), 3 (125 °C for 30 min), 4 (125 °C for 45 min), 5 (125 °C for 60 min), 6 (135 °C for 15 min), 7 (135 °C for 30 min), 8 (135 °C for 45 min), 9 (135 °C for 60 min), 10 (145 °C for 15 min), 11 (145 °C for 30 min), 12 (145 °C for 45 min), 13 (145 °C for 60 min).

N: The concentration of the compound of all treatments was below the odor threshold.

**Figure 1:**
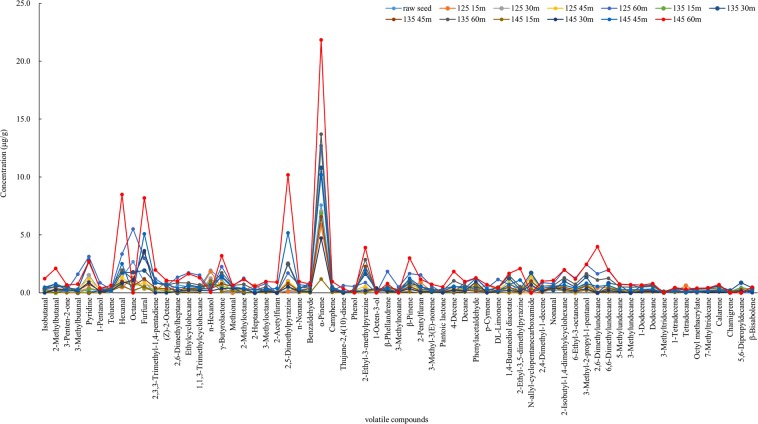
Effect of temperature and time on volatile compounds in sunflower seeds roasted from 125 to 145 °C for 15, 30, 45 and 60 min.

The major volatile components detected by GC-MS analysis were α-pinene, β-pinene, heterocyclic compounds (2-ethyl-3-methylpyrazine, 2,5-dimethylpyrazine, 2-ethyl-3,5-dimethylpyrazine pyridine), aldehydes (2-methylbutanal, furfural, hexanal, phenylacetaldehyde), hydrocarbons (octane, 2-isobutyl-1,4-dimethylcyclohexane, 6,6-dimethylundecane), alcohol (3-methyl-2-propyl-1-pentanol) and γ-butyrolactone (Fig. 1). While the main volatile compounds in raw sunflower seed were α-pinene, hexanal, furfural, octane, γ-butyrolactone.

The predominant compounds generally which are known to contribute to typical roast and nutty aroma presented in roasted sunflower seeds were 2,3-dimethylpyrazine, 2-ethyl-3-methylpyrazine, 2,5-dimethylpyrazine, 2-ethyl-3,5-dimethylpyrazine, and 2,6-diethyl-3-methylpyrazine. Those compounds were formed by Maillard reaction, a similar result was found in the previous study^14^. 2,5-Dimethylpyrazine was formed by heating L-threonine. Methylpyrazine and ethylpyrazine were the products or intermediates of decarbonylation followed by dehydration of heating L-serine^15^. 2-Acetylpyrrole was also known to be produced by Maillard reaction which was correlated with the roasty flavor. The minimum reaction time to form 2,3-dimethylpyrazine was 45 min, it corresponded to a temperature of at least 145 °C. The concentrations of 2,3-dimethylpyrazine were 0.55 and 1.30 μg/g at 145 °C for 45 and 60 min, respectively. 2-Ethyl-3,5-dimethylpyrazine, 2,5-dimethylpyrazine, 2-ethyl-3-methylpyrazine increased with time and temperature. 2,5-Dimethylpyrazine ranged from 0.20 (raw material) to 10.19 μg/g (145 °C for 60 min), 2-ethyl-3-methylpyrazine concentration increased from 0.14 (raw material) to 3.89 μg/g (145 °C for 60 min), 2-ethyl-3,5-dimethylpyrazine was formed at 125 °C for 30 min (0.19 μg/g). The minimum reaction time to produce 2,6-diethyl-3-methylpyrazine was 45 min, corresponding to a temperature of 125 and 135 °C, minimum reaction time was 30 min at 145 °C. The results indicated that the thermal processing of the seeds was necessary for the formation of those compounds, These pyrazines were also reported in the previous study concerning the typical aroma of other roasted seed, for example, pumpkin seed^14^, perilla seeds^16^. The results in the previous reference reported that the minimum temperature required to form these compounds in roasted pumpkin seeds was 100 °C, the temperature higher than 150 °C was necessary to form pyrazines.

Pyrazines have been reported to be some of the most important flavor compounds in the roasted nuts, whereas aldehydes were responsible for dominating off-flavors produced during process and storage^17^. 14 aldehydes were detected in the roasted sunflower seeds. The compounds derived from Strecker degradation, such as isobutanal, 2-methylbutanal, 3-methylbutanal, and phenylacetaldehyde increased significantly, especially roasting for 60 min at three temperature. 2-Methylbutanal and 3-methylbutanal were produced from isoleucine and leucine. They were known as a pleasing odor in some roasted foods^18^. Benzaldehyde was supposed to be a degradation product of phenylalanine^19^, showing similar behavior. Benzaldehyde contributed to bitter aroma^13^, showed higher contents in the raw and seeds roasted at the higher temperature and time (60 min). Similar to our results, the concentration of benzaldehyde was higher than other volatiles in raw almonds^18^. Other aldehydes found in the roasted seeds were produced from lipid oxidation, especially hexanal, pentanal, and nonanal showed a large increase in concentrations at the higher temperature and time. On account of their sensory attributes, those compounds were responsible for the fresh and slightly green flavor of the roasted sunflower seeds. Furfural which was the main furan compound found in the roasted sunflower seeds increased with the temperature and time, 5-methyl-2-furfural existed in the sunflower seeds roasted at 145 °C for 45 and 60 min. Methional and phenylacetaldehyde showed a similar behavior of furfural which increased with the temperature and time. Trans-2-octenal was only observed at 145 °C for 45 and 60 min.

Four alcohols were identified in the roasted sunflower seeds including 1-pentanol, hexanol, 1-octen-3-ol, and 3-methyl-2-propyl-1-pentanol. 1-Pentanol increased significantly (P < 0.05) at 60 min of three temperatures. 1-Octen-3-ol decreased significantly (P < 0.05) as the temperature and time grew. Hexanol decreased greatly (P < 0.05) with the increase of roasting temperature and time, the highest concentration was observed at 125 °C. Meanwhile, the amount of the corresponding hexanal increased significantly (P < 0.05), its probable mechanism may be the result of oxidation of the alcohol to the corresponding aldehyde. Whereas the amount of 3-methyl-2-propyl-1-pentanol increased up to 60 min at three temperature.

Toluene decreased up to 30 min, after that increased during roasting with the amount of 0.24 to 0.63 μg/g, the decrease may be due to the original toluene exist in the raw seed volatilized during thermal treatment. It was previously identified to have a negative effect on roasted and peanut aromas^20,21^; as it is responsible for a paint aroma.

Generally, furans are normally responsible for the caramel-like odor of heated carbohydrates^22^. With the exception of furfural, There were other furan compounds found in sunflower seeds including 2-acetylfuran, 2,5-dimethyltetrahydrofuran, and 2-pentylfuran. These compounds represented from 0 up to 0.91, 0 up to 0.51, and 0.33 up to 1.53 μg/g in the roasted sunflower seeds with the increasing time.

Most of the volatiles increased in the roasted sunflower seeds were already present in raw seeds. 41 volatiles were not present in the raw seed that formed during thermal treatment, including 20 hydrocarbons, 6 easters, 5 ketones, 3 furans, 2 pyrazines, 2 aldehydes, 1 phenol, 1 pyrrole, 1 ether. Based on comparing the key odor compounds in roasted and raw samples, only a quite limited number of important odor compounds in roasted sunflower seed, such as 2-ethyl-3,5-dimethyl-pyrazine, 2,3-dimethylpyrazine, 2-acetylpyrrole, 2,5-dimethyltetrahydrofuran, 5-methyl-2-furfural were clearly formed during roasting. On the other side, 2-methylbutanal, 3-methylbutanal, furfuran, 2,5-dimethylpyrazine, α-pipene, 1-octen-3-ol, benzaldehyde were already present in considerable contents in the raw samples. The results showed that α-pinene was by far the most abundant odorant (7.05 to 21.85 μg/g in roasted seed and 7.57 μg/g in the raw seed) followed by 2,5-dimethylpyrazine, hexanal, and furfural in the roasted seed with amounts of 0.20 to 10.19, 1.35 to 8.49 and 0.95 to 8.18 μg/g, respectively. Furfural and hexanal in the raw seed with amounts of 1.35 and 0.95 μg/g, respectively (Table 1). β-Pinene is an organic compound of the terpene class which is one of the two isomers of pinene, it has a woody-green pine-like smell. β-Pinene increased with the increasing temperature and time with the amount of 0.76 to 3.00 μg/g. Table 2 showed the key odorants content obtained from RSF at 125, 135 and 145 °C of roasting for 15, 30, 45 and 60 min. When the concentration of the volatile compound is higher than its threshold, it is accepted that the volatile compound is perceived. The concentration of 2,5 dimeththyl-pyrazine, 2,3-dimethylpyrazine, 2-ethyl-3-methylpyrazine, 3-ethyl-2,5-dimethylpyrazine, 2-ethyl-3,5-dimethylpyrazine detected in the RSF were superior to their threshold, those compounds were considered representative of the active compound, suggesting that nutty, roast characteristics might be responsible for the aroma of roasted sunflower seeds. 2,5 dimeththylpyrazine showed enough concentration to be detected at 135 °C for 30 and 60 min and 145 °C for 45 and 60 min in the RSF. 2-Ethyl-3-methylpyrazine showed the high concentration in all treatments except 145 °C for 60 min in the RSF, and it was thus significantly responsible for the characteristic nutty aroma of roasted sunflower seed. As a result of the low threshold value of 2-methylbutanal, it showed the major potential to be responsible for almond and malt aroma in the RSF. Phenylacetaldehyde had lower threshold value, the concentrations in all treatment of RSF were superior to its threshold, it suggested to be the major role in the flowery and sweet flavor. Hexanal and nonanal were responsible for the off-flavor because the concentrations of those compounds in the RSF were superior to its threshold, hexanal can be detected at all the treatments in RSF. Nonanal was not detected at 125 °C for 30 and 45 min, 135 °C for 30 and 60 min, 145 °C for 60 min. Heptanal was not detected at all treatment. Furfuryl alcohol along with furfural presented in many fruits, tea, coffee^23^, and their flavor characteristics was known as sweet, bread-like and caramellic. They are formed during the acid hydrolysis or heating of polysaccharides containing hexoses or pentoses^23^. Based on the results, the major contributors to bread, almond, sweet aroma came from furfuryl alcohol and furfural. The high content of pyridine was observed at a higher temperature, as the concentration superior to its odor threshold, it was possibly responsible for the burnt aroma. Moon and Shibamoto^24^ reported that γ-butyrolactone generated from chlorogenic acid degradation formed more at high roasting temperature. γ-Butyrolactone had higher concentration superior to the odor threshold value, which was responsible for creamy odor. a-Pinene was the major volatile compound detected in the RSF, the concentration was far in excess of the odor threshold, and possibly be responsible for the pine aroma.

### Principal component analysis (PCA) and agglomerative hierarchical clustering (AHC) analysis

**Figure 2:**
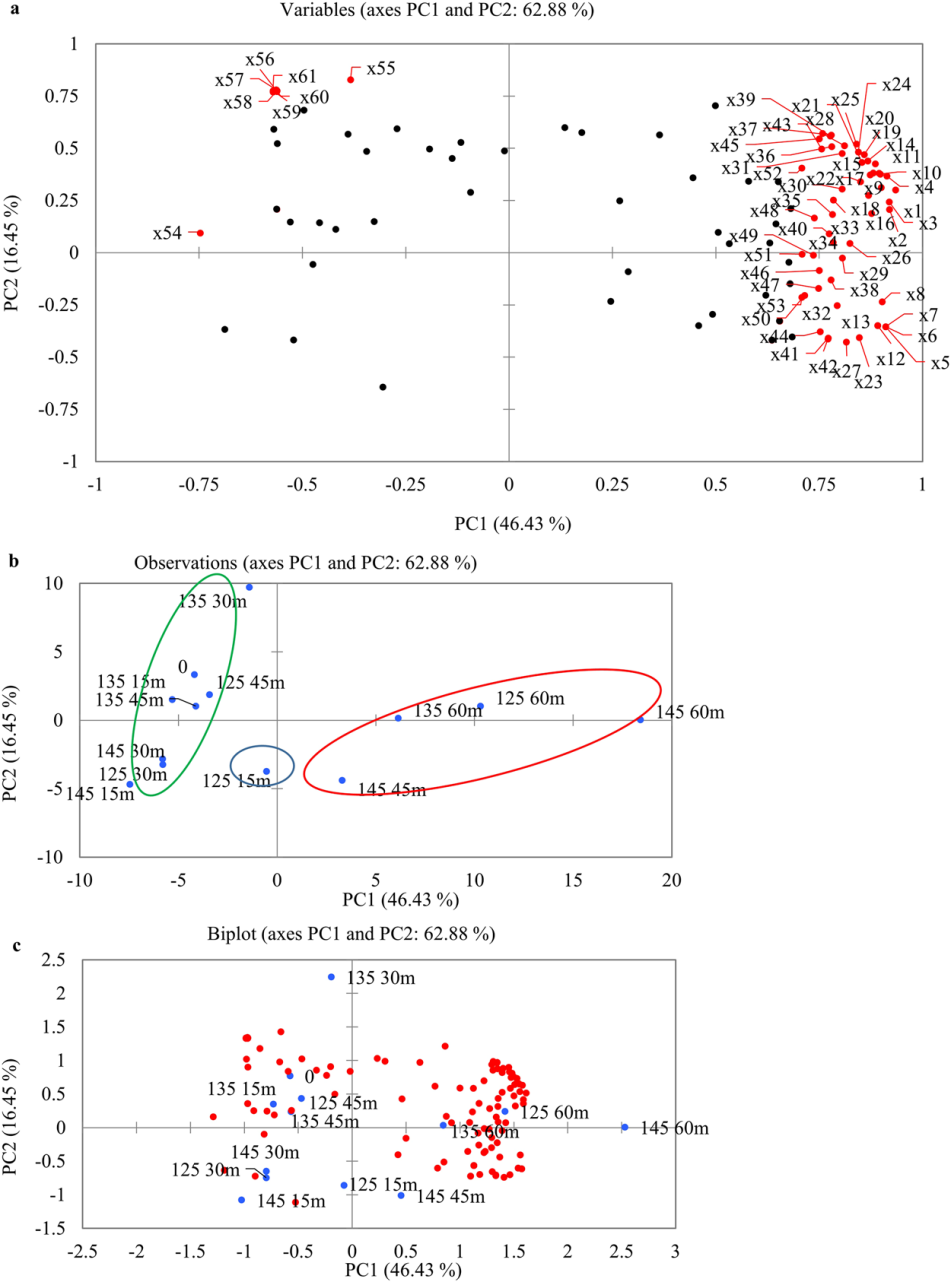
Principal component analysis on 114 volatile compounds determined by HS-SPME-GC/MS for different roasting time. (**a**) PCA loading plot with compound codes. Compound codes are explained in Table 3, significant factor loading value of each compound in Table 3 is higher than or equal to 0.7. The red circle in panel (**a**) represents the loading value higher than or equal to 0.7. (**b**) PCA score plot with sample labeling (**c**) PCA biplot with sample labeling.

Chemometrics is the methods using mathematical and statistical to improve the understanding of chemical information and to correlate quality parameters to analytical instrument data^25^. PCA and AHC methods were used to evaluate the flavor profile of roasted sunflower seeds. PCA for volatile compounds was represented on the loading plot and score plot which was obtained from the Spearman correlation data matrix of variables (Fig. 2a,b). As a result of PCA, the first PCs had the highest eigenvalue of 52.928 and explained 46.43% of the total variability of the data set. The second principal component had an eigenvalue of 18.757 and showed 16.45% of the total variability of the data set. Thus the first two principal components contributed to the total variability of 62.88%. Factor loadings are squared correlation between variables and factors that have been derived from PCA^26^. These numbers represent the significant contribution of volatiles to the total variability. The significant factor loading value higher than or equal to 0.7^27^ was applied to identify the most significant variable compound contributing to each PC(s). The PCA loading plot (Fig. 2a) with compound codes which are explained in Table 3 indicated that compounds formed during roasting process drive the separation. The factor loadings values higher than 0.7 were presented in Table 3. Loadings close to −1 or 1 indicate that the variable strongly influences the component. Loadings close to 0 indicate that the variable has a weak influence on the component^26^. The PC1 was highly positively contributed by hydrocarbons, ketones and as well as pyrazines (i.e. γ-butyrolactone (x1), 6,6-dimethylundecane (x2), β-pinene (x3), decane (x4), 2,6-dimethylundecane (x5), 3-methylundecane (x6), 4-methyl-5-propylnonane (x7), 2-ethylhexyl acrylate (x8), 2,4-dimethyl-1-decene (x9), 3-methyloctane (x10), n-nonane (x11), 1-tert-butoxy-6-methylcyclohexene (x12), (Z)-7-methyl-5-undecene (x13), 2-isobutyl-1,4-dimethylcyclohexane (x14), ethyl-cyclohexane (x15), 2,3,3-trimethyl-1,4-pentadiene (x16), β-phellandrene (x17), hexanal (x18), 3-methyl-2-propyl-1-pentanol (x19), 3-methyl-3(E)-nonene (x20), 1,1,3-trimethylcyclohexane (x21), α-pinene (x22)). While chamigrene (x55), 2,6-dimethyloctadecane (x56), 2,4-dimethyldodecane (x57), N-allyl-cyclopentanecarboxamide (x58), 2,6-diethyl-3-methylpyrazine (x59), 3-methyl-2(E)-undecene (x60), 5,6-dipropyldecane (x61), and 3-penten-2-one (x62) attributed significantly towards PC2. 1-Octen-3-ol (x54) was closely located on the negative side of PC1.

**Table 3:** Varimax rotated factor loadings of the significant principal components (PCs).

PCA code	Variables	PC1	PCA code	Variables	PC1
x1	γ-Butyrolactone	0.935	x33	6-Ethyl-3-octanone	0.784
x2	6,6-Dimethylundecane	0.920	x34	1-Pentanol	0.784
x3	β-Pinene	0.920	x35	1,3,5-Trimethylcyclohexane	0.782
x4	Decane	0.914	x36	Calarene	0.780
x5	2,6-Dimethylundecane	0.911	x37	7-Methyltridecane	0.779
x6	3-Methylundecane	0.911	x38	(Z)-2-Octene	0.778
x7	4-Methyl-5-propylnonane	0.911	x39	Octyl methacrylate	0.777
x8	2-Ethylhexyl acrylate	0.902	x40	2-Pentylfuran	0.774
x9	2,4-Dimethyl-1-decene	0.900	x41	2-Ethylhexyl mercaptoacetate	0.772
x10	3-Methyloctane	0.898	x42	2-Heptanone	0.771
x11	n-Nonane	0.895	x43	D-Limonene	0.756
x12	1-*tert*-Butoxy-6-methylcyclohexene	0.892	x44	3,7-Dimethyldecane	0.752
x13	(Z)-7-Methyl-5-undecene	0.892	x45	1-Tetradecene	0.750
x14	2-Isobutyl-1,4-dimethylcyclohexane	0.885	x46	2-Methyloctane	0.750
x15	Ethyl-cyclohexane	0.881	x47	*trans*-2,2-Dimethyl-3-decene	0.748
x16	2,3,3-Trimethyl-1,4-pentadiene	0.877	x48	2-Ethyl-3-methylpyrazine	0.738
x17	β-Phellandrene	0.873	x49	2,5-Dimethylpyrazine	0.736
x18	Hexanal	0.869	x50	Nonanal	0.714
x19	3-Methyl-2-propyl-1-pentanol	0.868	x51	Methional	0.709
x20	3-Methyl-3(E)-nonene	0.859	x52	Isovaleraldehyde	0.708
x21	1,1,3-Trimethylcyclohexane	0.853	x53	Camphene	0.708
x22	α-Pinene	0.850	x54	1-Octen-3-ol	-0.747
x23	2,5-Dimethyltetrahydrofuran	0.847			PC2
x24	Dodecane	0.845	x55	Chamigrene	0.828
x25	1,4-Butanediol diacetate	0.840	x56	2,6-Dimethyloctadecane	0.780
x26	2-Ethyl-3,5-dimethylpyrazine	0.824	x57	2,4-Dimethyldodecane	0.780
x27	γ-Valerolactone	0.816	x58	N-allyl-cyclopentanecarboxamide	0.776
x28	5-Methylundecane	0.811	x59	2,6-Diethyl-3-methylpyrazine	0.775
x29	p-Cymene	0.806	x60	3-Methyl-2 (E)-undecene	0.772
x30	Benzaldehyde	0.805	x61	5,6-Dipropyldecane	0.771
x31	1-Dodecene	0.805	x62	3-Penten-2-one	0.705
x32	2,5-Dimethylhexane	0.793			

The score plot of volatiles generated from the comparison of the first two PCs (Fig. 2b). Sunflower samples were separated based on roasting temperatures and times. Volatile profiles in the highest temperature and late roasting times (145 °C, 45 and 60 min) separated from early roasting times (15 and 30 min). Along the PC1, late roasting times clustered on the right side while early roasting times clustered on the left side. PC1 and PC2 clearly separated treatments into three groups. Cluster I consisted of four treatments (125 °C for 60 min, 135 °C for 60 min, 145 °C for 45 min, 145 °C for 60 min) due to its positive correlation of volatile compounds on PC1. Cluster II including a single treatment 125 °C for 15 min, cluster III consisted eight treatments (0, 125 °C for 30 and 45 min, 135 °C for 15, 30 and 45 min, 145 °C for 15 and 30 min). Figure 2c showed PCA biplot which combined PCA score plot and loading plot.

**Figure 3:**
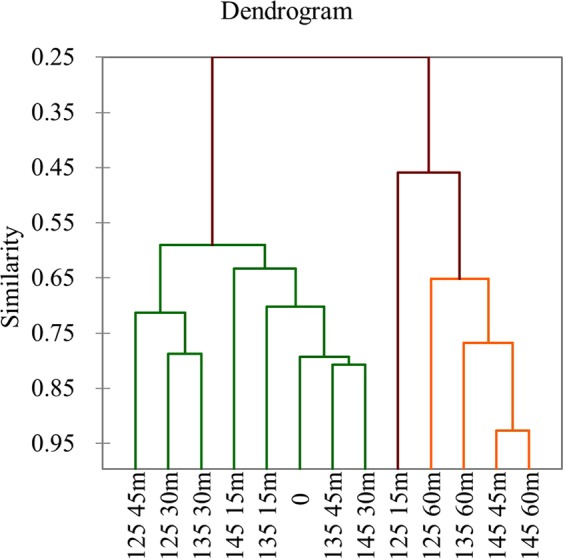
Dendrogram showing Agglomerative hierarchical clustering (AHC). The volatile compounds contents of roasted sunflower seeds were analyzed using AHC.

AHC also gave an overview of similarities and differences among the treatments. These results were following the PCA analysis. AHC dendrogram of 114 volatile compounds was shown in Fig. 3. Dendrogram generated from hierarchical clustering was to assess the relationship between treatments^28^. The data sets were grouped into three clusters, whereby all the treatments of the close similarity were grouped together.

## Conclusions

DVB/CAR/PDMS absorbed a large number of volatile compounds in roasted sunflower seeds, HS-SPME combined with GC-MS was used to evaluate the dynamic change of flavor profile during roasting sunflower seed. 114 volatiles were identified and quantified in the roasted sunflower seeds. The influence of roasting on flavor development varies depending on the temperature and time. The typical aroma compounds from Maillard reaction were formed at higher temperature and time, such as terpenes, pyrazines, and aldehydes. 2-Ethyl-3-methylpyrazine, 2,5-dimethylpyrazine, 2,3-dimethylpyrazine, and 2-ethyl-3,5-dimethylpyrazine which contribute to roasty and nutty flavor were found in higher concentration in the roasted sunflower seeds. The undesirable flavor notes, such as hexanal, nonanal, and pyridine were produced more in the sunflower seed roasted at three temperature for long-term. 125 °C for 45 min was found to be a better condition for roasting sunflower seeds, which gave the lowest off-flavor and burnt tastes.

PCA and AHC analysis were used for discriminating the variation among the 13 treatments. Three groups were separated on the basis of PCA and AHC analysis. Cluster I consisted of four treatments (125 °C for 60 min, 135 °C for 60 min, 145 °C for 45 min, 145 °C for 60 min) due to its positive correlation of volatile compounds on PC1. Cluster II including a single treatment 125 °C for 15 min, cluster III consisted eight treatments (0, 125 °C for 30 and 45 min, 135 °C for 15, 30 and 45 min, 145 °C for 15 and 30 min).

## Materials and Methods

### Material

Sunflower seeds harvested in 2017 were purchased from the local market, Thailand. The seeds were collected in aluminum foil bags and stored at 4 °C. By removing small, shriveled and broken seeds, good quality sunflower seeds were selected for further use.

### Reference standards and reagents

Ethyl decanoate at 100 μg/2 g of sunflower seed (ethyl decanoate 1 mg/ml in 10% methanol) was used as an internal standard. The following references were used for identification experiments: isobutanol, 2-methylpropanal, 3-methylbutanal, 3-penten-2-one, 1-pentanol, 3-hydroxy-2-butanone, hexanal, n-hexanol, 2,5-dimethylhexane, furfural, 1-octen-3-ol, γ-butyrolactone, methional, 2-methyloctane, nonanal, L-carvone, benzaldehyde, heptanal, trans-2-heptenal, trans-2-octenal, camphene, phenol, 2-heptanone, p-cymene, 2-acetylfuran, 2-acetyl-3-methylpyrazine, 2,5-dimethylpyrazine, 2-ethyl-3-methylpyrazine, 2-methlypyrazine, 2-ethylpyrazine, 2,3-diethyl-5-methylpyrazine, pyridine, 2-ethyl-3,5-dimethylpyrazine, 2-acetylpyrrole, 2,3-dimethylpyrazine, γ-valerolactone, 2,5-dimethyltetrahydrofuran, 2-pentylfuran, phenylacetaldehyde, D-Limonene, 2-ethylhexyl acrylate, β-phellandrene, α-pinene, β-pinene, ethyl nonanoate, and ethyl octanoate. All reference standards were prepared in acetone at a concentration of 0.5% (weight/volume). n-Alkanes (C6-C26) were used as standards for retention index calculation. All the reference standards were obtained from Sigma-Aldrich, USA. All reagents were obtained from Fisher Scientific (Thermo Fisher Scientific, Waltham, MA).

### Roasting process

Sunflower seeds were roasted by an electric forced air oven (Model UF55, Memmert, Thailand) at 125, 135 and 145 °C for 15, 30, 45, 60 min. The roasted sunflower seeds were cooled to ambient temperature, shelled and ground by an electric grinder (Panasonic, Japan) and stored in a sealed aluminum foil bag at −20 °C for further analysis.

### Flavor extraction

For extraction of volatiles, the ground roasted sunflower seeds (2 g) was placed into headspace extraction vial, with 100 μg/2 g ethyl decanoate of sunflower seed (ethyl decanoate 1 mg/ml in 10% methanol) internal standard, prior to sealing with caps. 1 μl C6-C26 n-alkanes mixture (100 μg/ml each in methanol) were analyzed under the same condition. The sample was equilibrated for 20 min at 60 °C in the HS of the vial. After the equilibration, a 50/30 μm DVB/CAR/PDMS SPME fiber (57348-U, Supelco) was exposed to the HS for 30 min at 60 °C. PDMS is used for non-polar analytes, DVB is for polar analytes, especially useful for pyrazines. The application of this fiber successfully identified the aroma compounds of roasted almond^29^ and roasted plantains^30^.

### Identification of the volatile flavor compounds

GC-MS system Agilent 7890A gas chromatograph (Agilent Technologies, USA) with a 5975C mass spectrometer was used for analysis. A 60 m × 0.25 mm × 0.25 μm DB-1 ms column was used for analytes separation. The analytes were desorbed to the hot injection port of GC for 20 min at 250 °C in a splitless mode. Helium was operated at a constant flow rate of 1.5 ml/min. The temperature program was 50 °C for 1 min, followed by 5 °C/min to 100 °C (5 min), 4 °C/min to 140 °C (5 min), 5 °C/min to 180 °C (2 min), and 10 °C/min to 250 °C (7 min). The MS source temperature was 230 °C, transfer line temperature was 225 °C, quadrupole temperature was 150 °C. The electron ionization energy was set at 70 eV, scan range, m/z 50–550. The reference standards were operated under the same GC-MS condition described previously, an injection volume of 0.2 μl reference standard mixtures was employed in split mode (split ratio 100:1). The temperature program for n-alkane mixture was 5 °C/min to 100 °C (5 min), 4 °C/min to 140 °C (5 min), 5 °C/min to 180 °C (2 min), 10 °C/min to 250 °C (7 min), 10 °C/min to 280 °C (5 min), and 5 °C/min to 300 °C (10 min).

The identification of volatile compounds was comparison mass spectra with reference standards. Volatile compounds without authentic standards were identified by comparing retention indexes and/or mass spectrum based on the NIST library (NIST 11, Version 2.0, Gaithersburg, USA). Retention index of each compound was calculated by the retention time of a series of C6-C26 n-alkanes. The relative concentration of each compound was calculated based on the area of the internal standard.

### Statistical analysis

PCA and AHC analysis were used to analyze the correlation between samples and variables and variables themselves. The results were analyzed by one-way analysis of variance (ANOVA) and differences among treatments were carried out using the Tukey’s range test with a 95% significance level (P < 0.05). All the statistical analyses were performed by XLSTAT version 2016.7 (Addinsoft, NY, USA).

## Data Availability

The data used to support the findings of this study are available from the corresponding author upon request.
